# Supplementary material for: Study of Biological Activities and ADMET-Related Properties of Salicylanilide-Based Peptidomimetics
Source: Int J Mol Sci. 2022 Oct 1;23(19):11648. doi: 10.3390/ijms231911648 (PMC9569995; doi:10.3390/ijms231911648)

Supplementary Materials

Study of Biological Activities and ADMET-Related Properties of Salicylanilide-based Peptidomimetics

Dominika Pindjakova [1], Eliska Pilarova [2], Karel Pauk [2], Hana Michnova [3], Jan Hosek [4], Pratibha Magar [2], Alois Cizek [3], Ales Imramovsky [2,*] and Josef Jampilek [1,5]

[1] Department of Analytical Chemistry, Faculty of Natural Sciences, Comenius University, Ilkovicova 6, 842 15 Bratislava, Slovakia
[2] Institute of Organic Chemistry and Technology, Faculty of Chemical Technology, University of Pardubice, Studentska 95, CZ-530 09 Pardubice, Czech Republic
[3] Department of Infectious Diseases and Microbiology, Faculty of Veterinary Medicine, University of Veterinary Sciences Brno, Palackeho tr. 1946/1, 612 42 Brno, Czech Republic
[4] Department of Pharmacology and Toxicology, Veterinary Research Institute, Hudcova 296/70, 621 00 Brno, Czech Republic
[5] Department of Chemical Biology, Faculty of Science, Palacky University Olomouc, Slechtitelu 27, 783 71 Olomouc, Czech Republic
* Correspondence: ales.imramovsky@upce.cz

Table of contents:

Table S1. Structures of benzylated precursors of diamides **2a–h** and triamides **5a–c** and their experimentally determined lipophilicity values……………………………………………………………2

Figure S1. Mutual comparison of experimentally determined values of log *k* (**A**), log $D_{6.5}$ (**B**) and log $D_{7.4}$ (**C**) of all final compounds **3a–f** and **6a–c**……………………………………………………………………………………..3

Figure S2. Comparison of experimentally determined values of log *k* of prepared compounds with predicted log *P* (ACD/Percepta) (**A**), log *P* (ChemBioDraw) (**B**) and Clog *P* (ChemBioDraw) (**C**) values……………………………..4

Figure S3. Comparison of experimentally determined values of log $D_{6.5}$ of prepared compounds with predicted log *P* (ACD/Percepta) (**A**), log *P* (ChemBioDraw) (**B**) and Clog *P* (ChemBioDraw) (**C**) values………………………5

Figure S4. Comparison of experimentally determined values of log $D_{7.4}$ of prepared compounds with predicted log *P* (ACD/Percepta) (**A**), log *P* (ChemBioDraw) (**B**) and Clog *P* (ChemBioDraw) (**C**) values……….……………...6

Table S2. In vitro antistaphylococcal, anti-enterococcal and antimycobacterial activities (MIC [μg/mL]), and *in vitro* cell viability (IC_{50} [μM] ± SD, *n* = 6) on human monocytic leukemia cell line (THP-1)………7

Copies of ^{1}H, ^{13}C and ^{19}FNMR spectra of compounds 3c – 3g (Figures S5 – S16) …………………………..………8

Table S1. Structures of benzylated precursors of diamides **2a–h** and triamides **5a–c** and their experimentally determined lipophilicity values (log *k*, log $D_{6.5}$, and log $D_{7.4}$).

No.	R¹	R²	log *k*	log $D_{6.5}$	log $D_{7.4}$
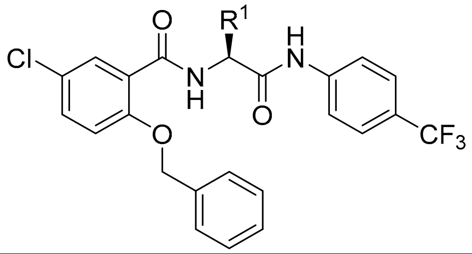					
**2a**	Me	–	1.450	1.489	1.474
**2b**	Pr	–	1.709	1.742	1.731
**2c**	iPr	–	1.683	1.711	1.697
**2d**	Bu	–	1.898	1.954	1.902
**2e**	*S*-Bu	–	1.668	1.714	1.689
**2f**	iBu	–	1.975	2.014	1.995
**2g**	Me-cHex	–	1.820	1.767	1.746
**2h**	Bn	–	1.852	1.889	1.871
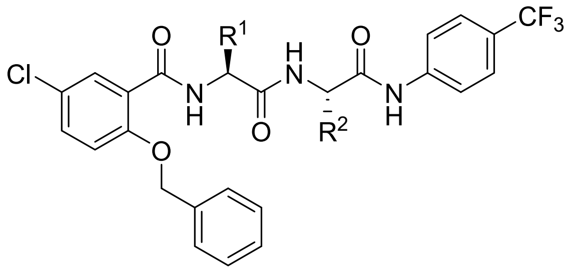					
**5a**	iBu	Bn	2.053	1.998	2.031
**5b**	Bn	iBu	2.049	1.994	2.027
**5c**	Bn	Bn	2.119	2.062	2.096

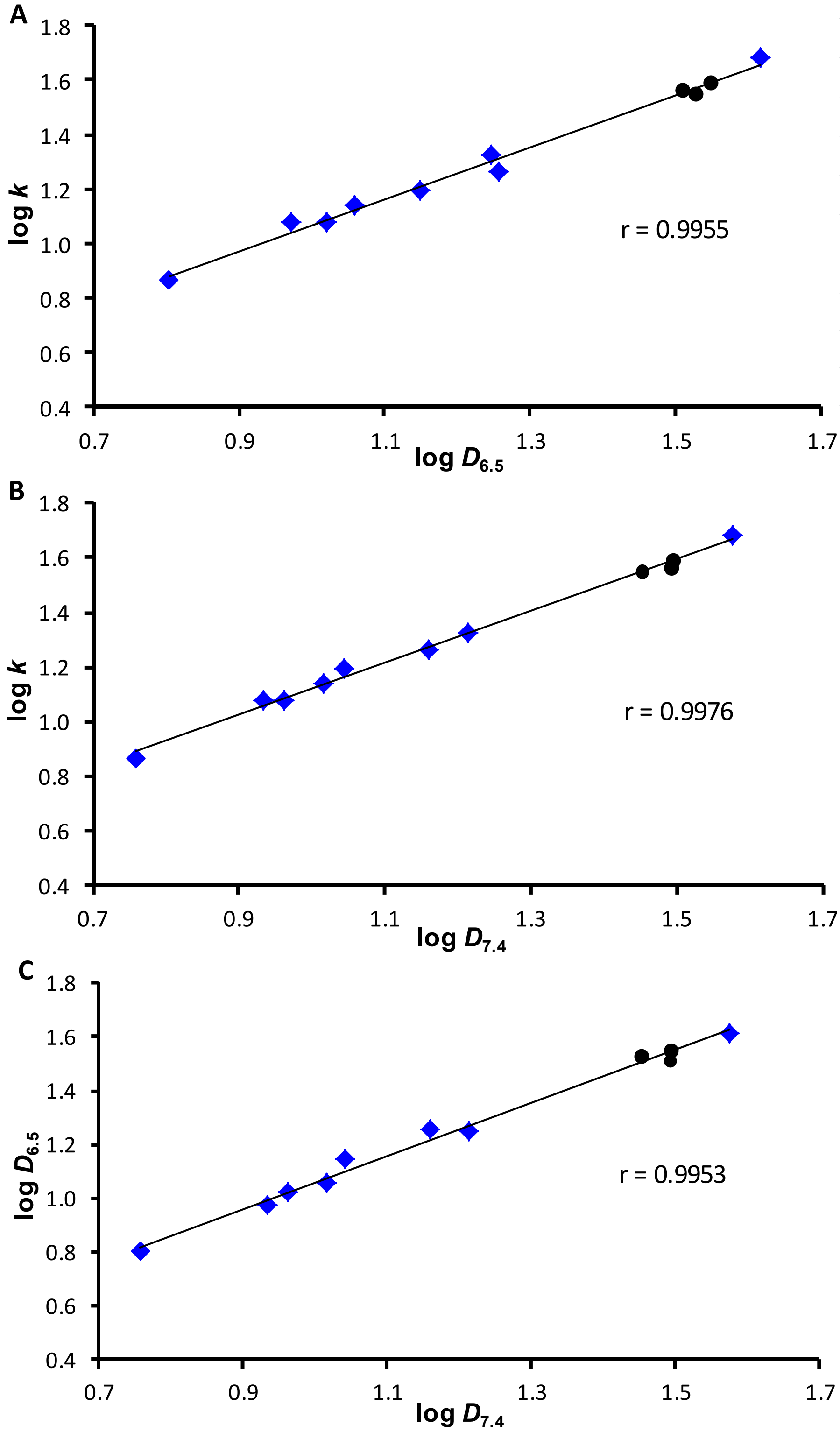

Figure S1. Mutual comparison of experimentally determined values of log *k* (**A**), log $D_{6.5}$ (**B**) and log $D_{7.4}$ (**C**) of all final compounds **3a–f** and **6a–c**. (blue rhombuses = diamides **3a–f**, black circle = triamides **6a–c**)

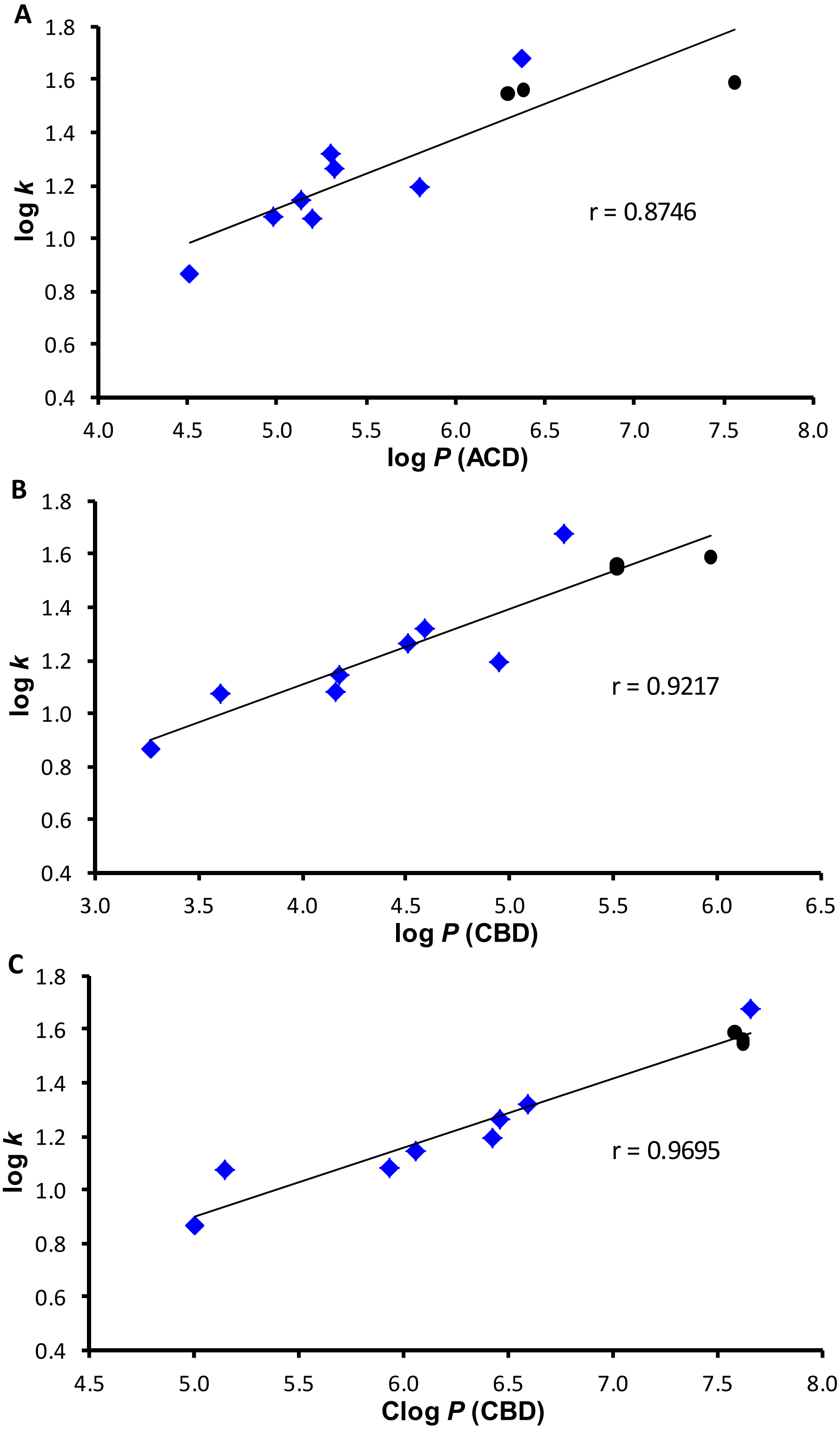

Figure S2. Comparison of experimentally determined values of log *k* of prepared compounds with predicted log *P* (ACD/Percepta) (**A**), log *P* (ChemBioDraw) (**B**) and Clog *P* (ChemBioDraw) (**C**) values. (blue rhombuses = diamides **3a–f**, black circle = triamides **6a–c**)

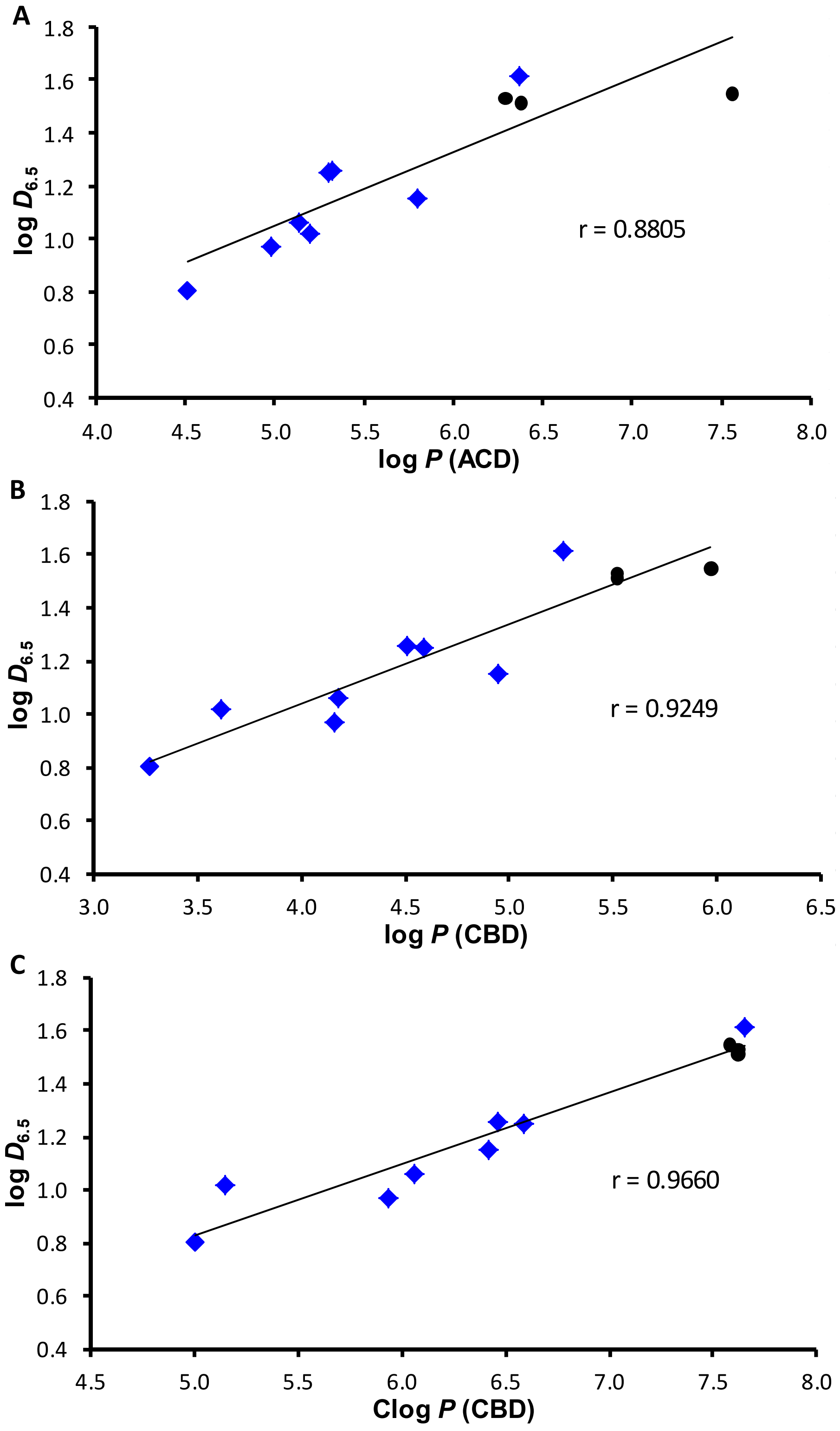

Figure S3. Comparison of experimentally determined values of log $D_{6.5}$ of prepared compounds with predicted log P (ACD/Percepta) (**A**), log P (ChemBioDraw) (**B**) and Clog P (ChemBioDraw) (**C**) values. (blue rhombuses = diamides **3a–f**, black circle = triamides **6a–c**)

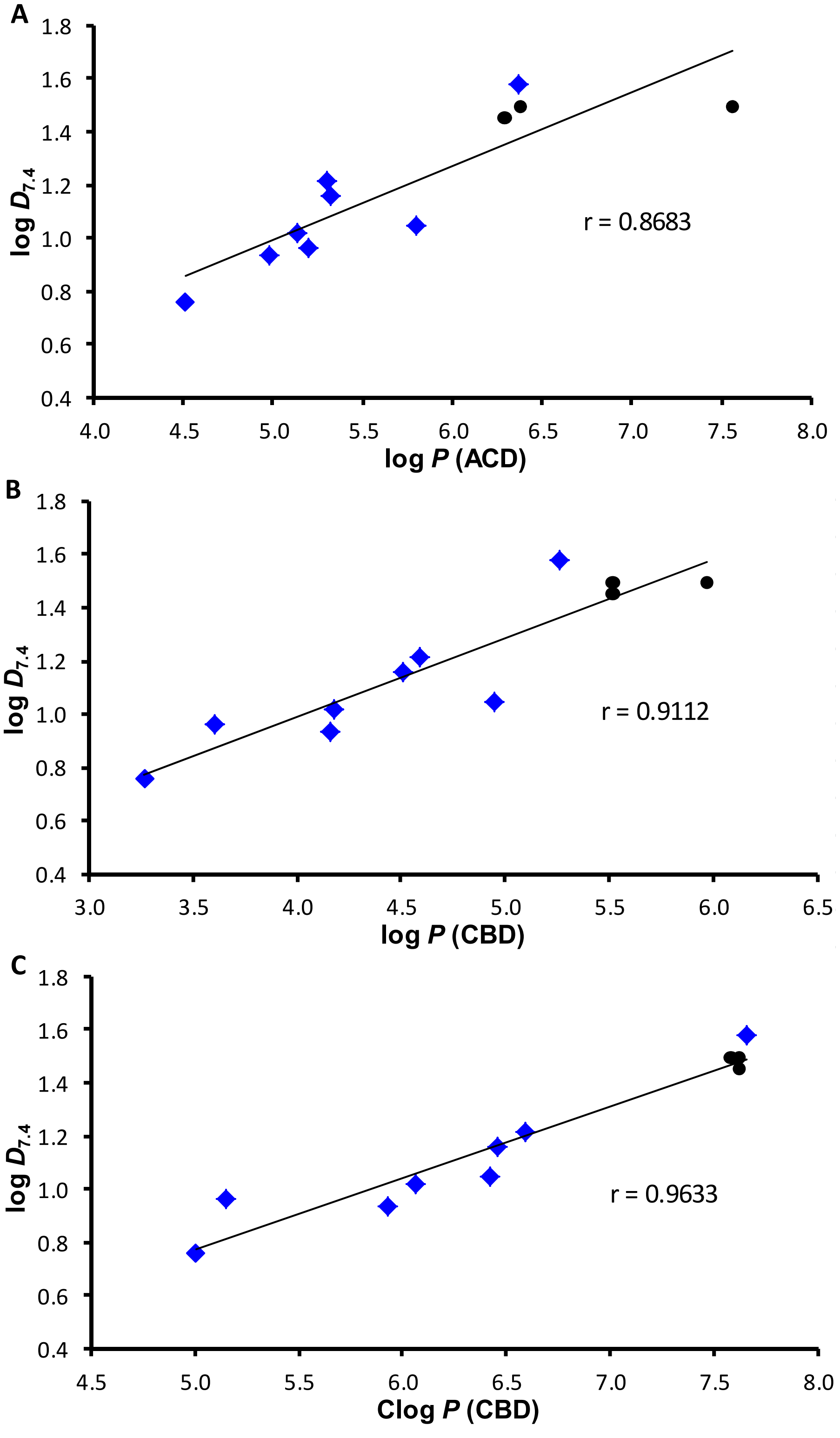

Figure S4. Comparison of experimentally determined values of log $D_{7.4}$ of prepared compounds with predicted log P (ACD/Percepta) (**A**), log P (ChemBioDraw) (**B**) and Clog P (ChemBioDraw) (**C**) values. (blue rhombuses = diamides **3a–f**, black circle = triamides **6a–c**)

Table S2. In vitro antistaphylococcal, anti-enterococcal and antimycobacterial activities (MIC [µg/mL]), and in vitro cell viability (IC_{50} [µM] ± SD, *n* = 6) on human monocytic leukemia cell line (THP-1).

No.	R[1] R[2]	MIC [µg/mL]										IC_{50} [µM] THP-1@10% FBS 24h
		SA	MRSA1	MRSA2	MRSA3	EF	VRE1	VRE2	VRE3	MT	MS	
**2a**	Me<br>–	>256	>256	>256	>256	>256	>256	>256	>256	>256	>256	>10
**2b**	Pr<br>–	>256	>256	>256	>256	>256	>256	>256	>256	>256	>256	>10
**2c**	iPr<br>–	>256	>256	>256	>256	>256	>256	>256	>256	>256	>256	>10
**2d**	Bu<br>–	>256	>256	>256	>256	>256	>256	>256	>256	>256	>256	>10
**2e**	*S*-Bu<br>–	>256	>256	>256	>256	>256	>256	>256	>256	>256	>256	>10
**2f**	iBu<br>–	>256	>256	>256	>256	>256	>256	>256	>256	>256	>256	>10
**2g**	Me-cHex<br>–	>256	>256	>256	>256	>256	>256	>256	>256	>256	>256	>10
**2h**	Bn<br>–	>256	>256	>256	>256	>256	>256	>256	>256	>256	>256	>10
**5a**	iBu<br>Bn	>256	>256	>256	>256	>256	>256	>256	>256	>256	>256	>10
**5b**	Bn<br>iBu	>256	>256	>256	>256	>256	>256	>256	>256	>256	>256	>10
**5c**	Bn<br>Bn	>256	>256	>256	>256	>256	>256	>256	>256	>256	>256	>10

SA = *Staphylococcus aureus* ATCC 29213; MRSA1–3 = clinical isolates of methicillin-resistant *S. aureus* SA 3202, SA 630 (National Institute of Public Health, Prague, Czech Republic), and 63718 (Department of Infectious Diseases and Microbiology, Faculty of Veterinary Medicine, University of Veterinary Sciences Brno, Czech Republic); EF = *Enterococcus faecalis* ATCC 29212, and vancomycin-resistant enterococci VRE1–3 = VRE 342B, VRE 368, VRE 725B, MT = *Mycobacterium tuberculosis* H37Ra/ATCC 25177; MS = *M. smegmatis* ATCC 700084; FSB = fetal bovine serum.

Copies of ^{1}H, ^{13}C and ^{19}FNMR spectra of most active compounds 3

Figure S5. Copy of ^{1}H NMR spectrum of 5-chloro-2-hydroxy-*N*-[(2*S*)-3-methyl-1-oxo-1-{[4-(trifluoromethyl)phenyl]amino}butan-2-yl]benzamide (**3c**).

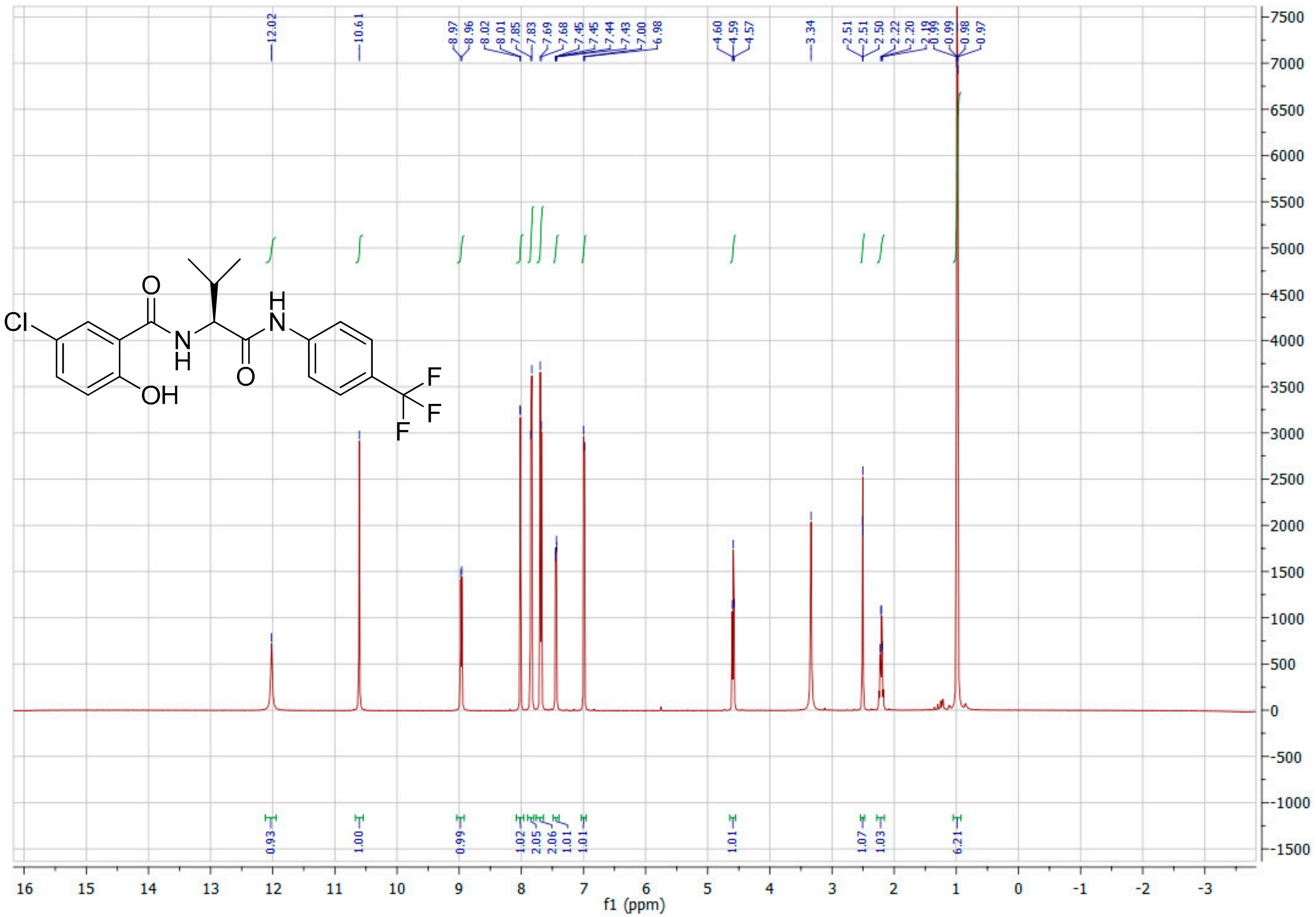

Figure S6. Copy of ^{13}C NMR spectrum of 5-chloro-2-hydroxy-*N*-[(2*S*)-3-methyl-1-oxo-1-{[4-(trifluoromethyl)phenyl]amino}butan-2-yl]benzamide (**3c**).

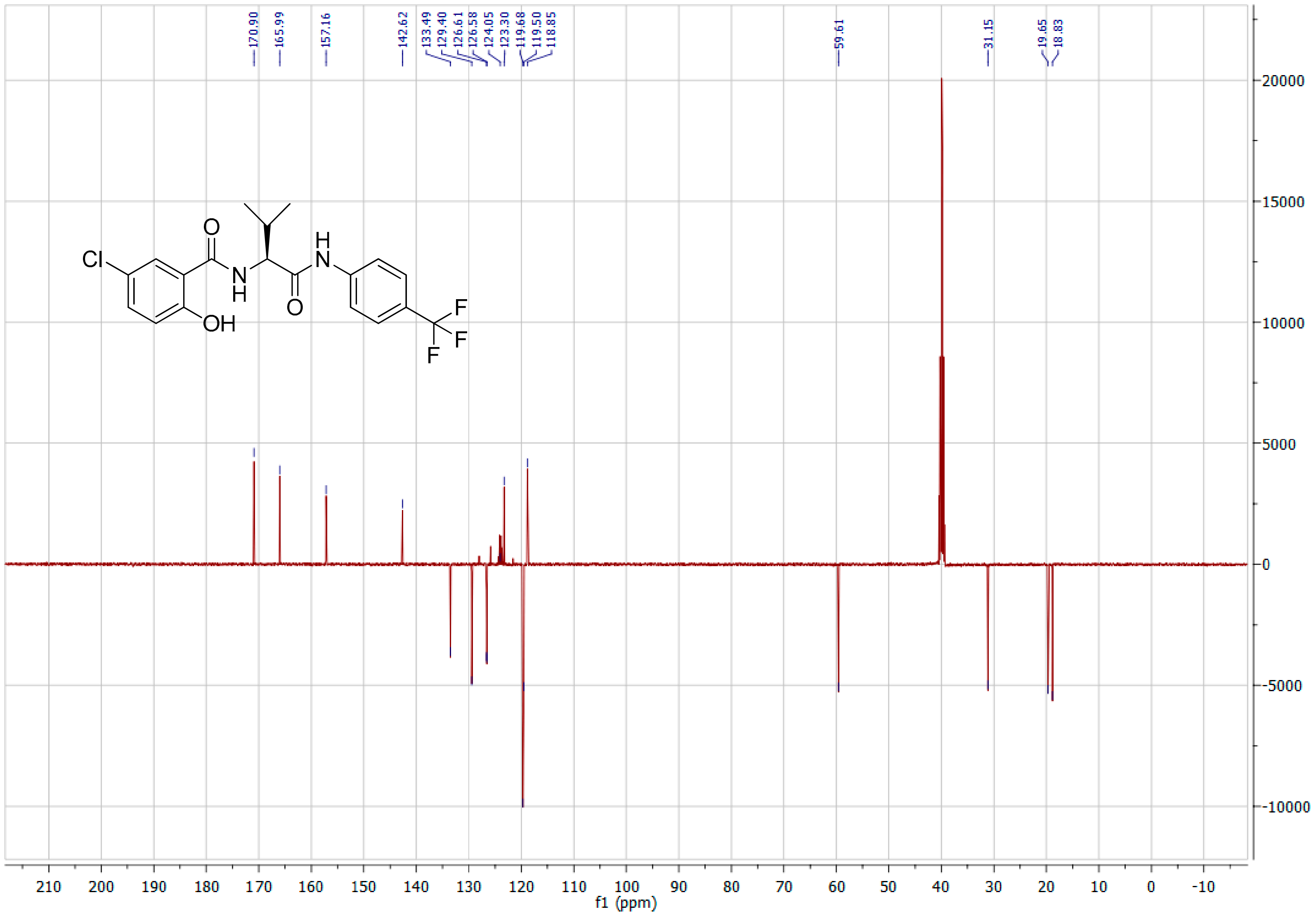

Figure S7. Copy of ^{19}F NMR spectrum of (*S*)-5-chloro-2-hydroxy-*N*-(3-methyl-1-oxo-1-((4-(trifluoromethyl)phenyl)amino)butan-2-yl)benzamide (**3c**)

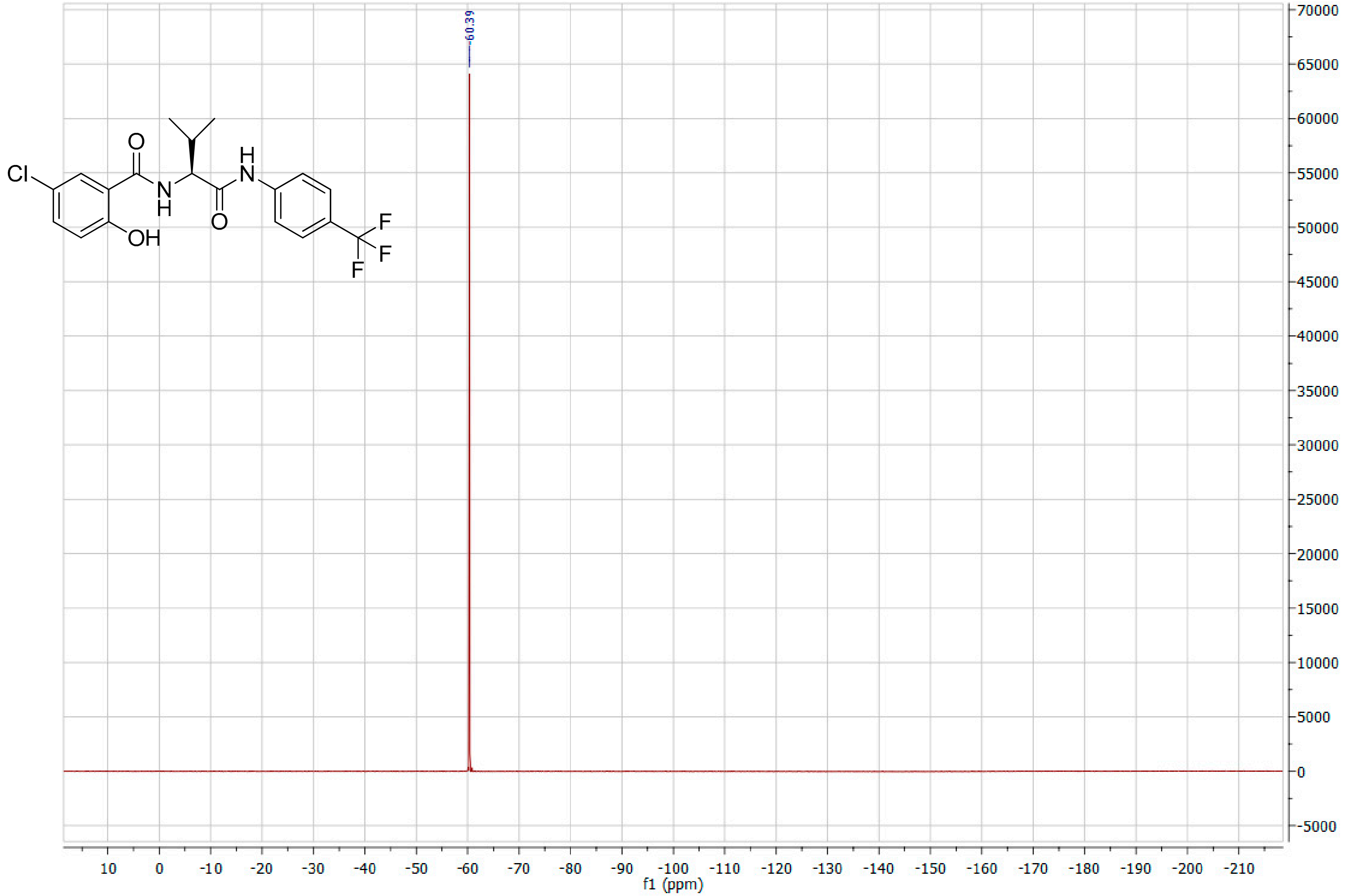

Figure S8. Copy of ¹H NMR 5-Chloro-2-hydroxy-*N*-[(2*S*)-1-oxo-1-{[4-(trifluoromethyl)phenyl]amino}hexan-2-yl]benzamide (**3d**)

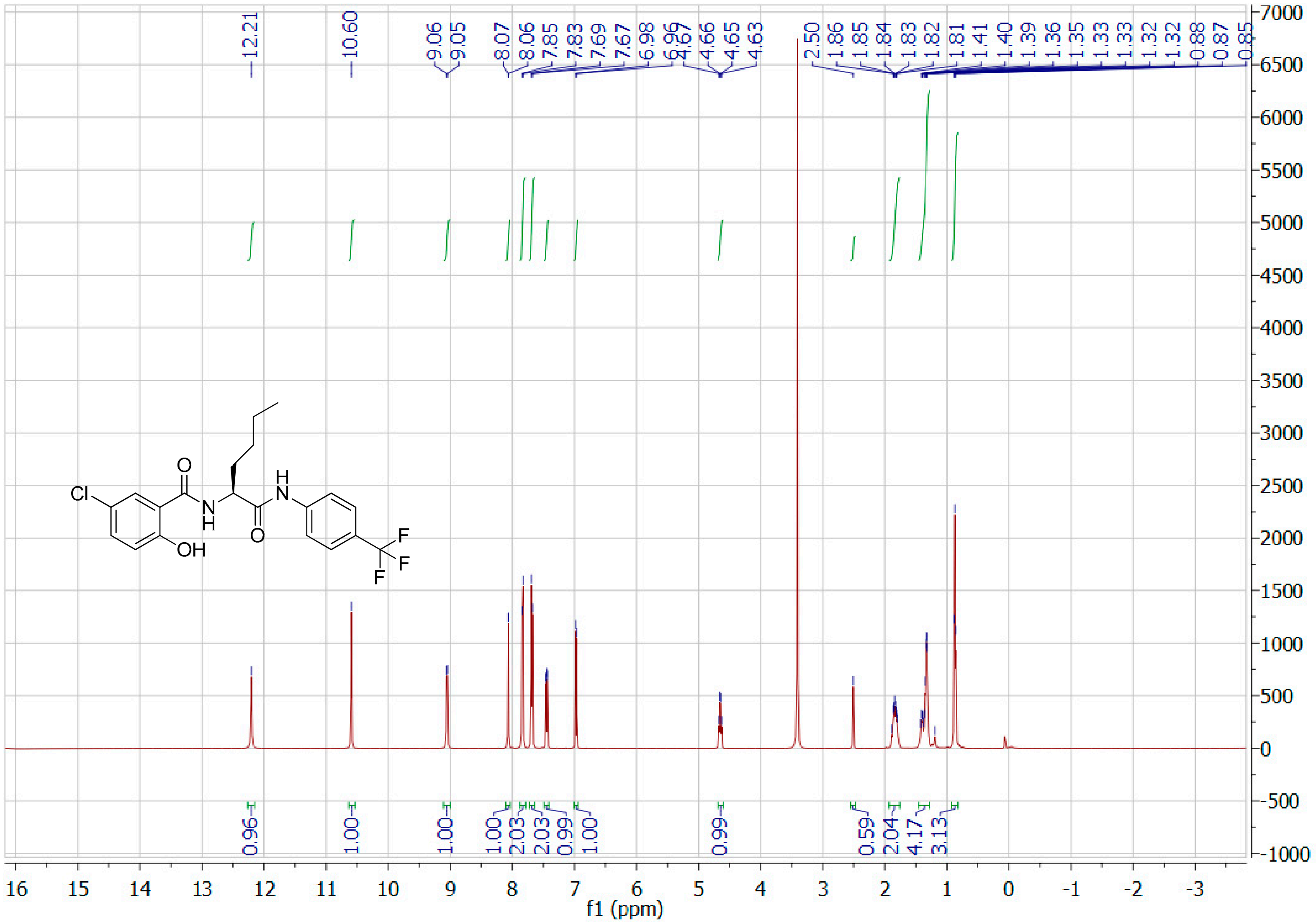

Figure S9. Copy of ^{13}C NMR 5-Chloro-2-hydroxy-*N*-[(2*S*)-1-oxo-1-{[4-(trifluoromethyl)phenyl]amino}hexan-2-yl]benzamide (**3d**)

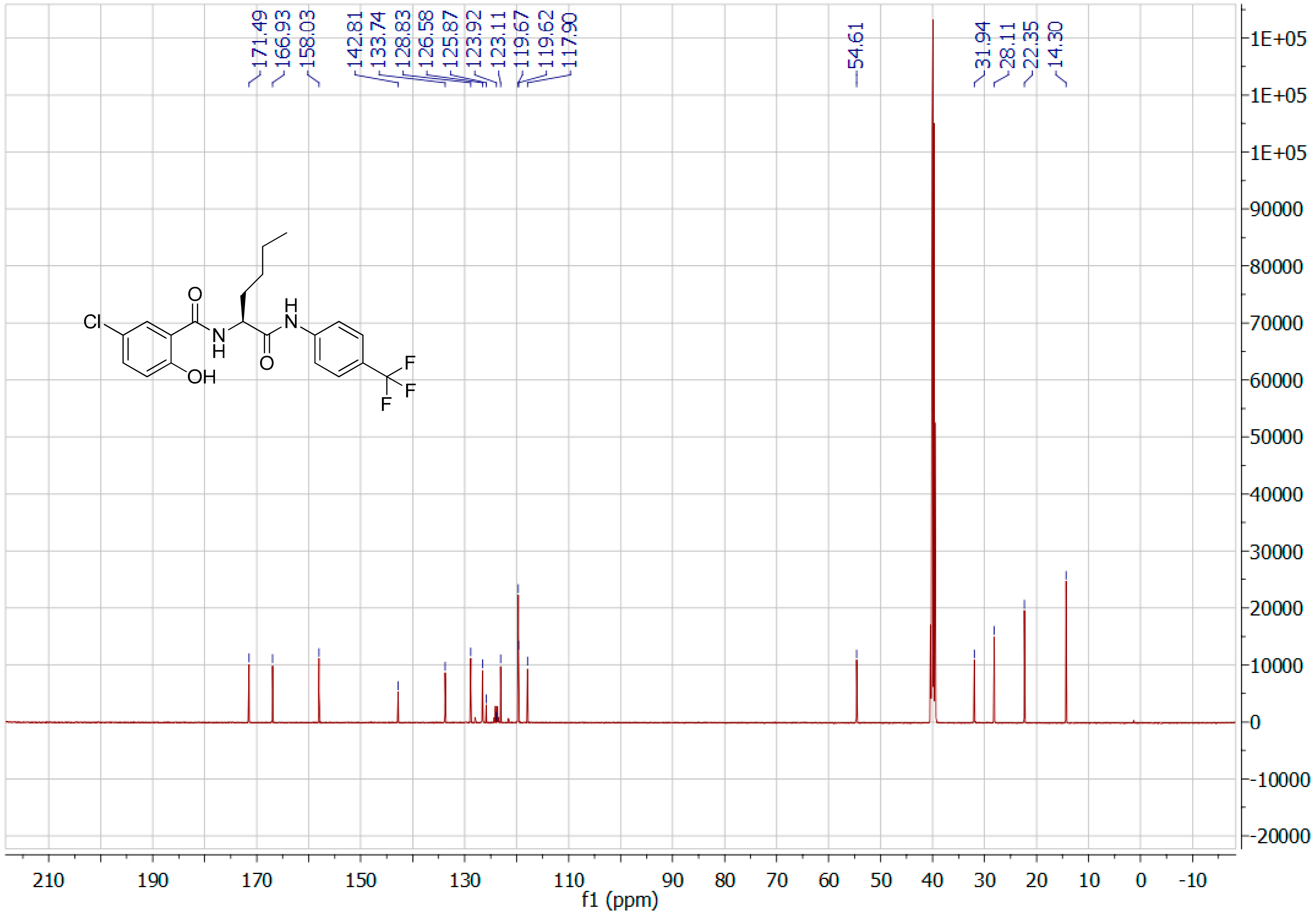

Figure S10. Copy of ^{19}F NMR 5-Chloro-2-hydroxy-*N*-[(2*S*)-1-oxo-1-{[4-(trifluoromethyl)phenyl]amino}hexan-2-yl]benzamide (**3d**)

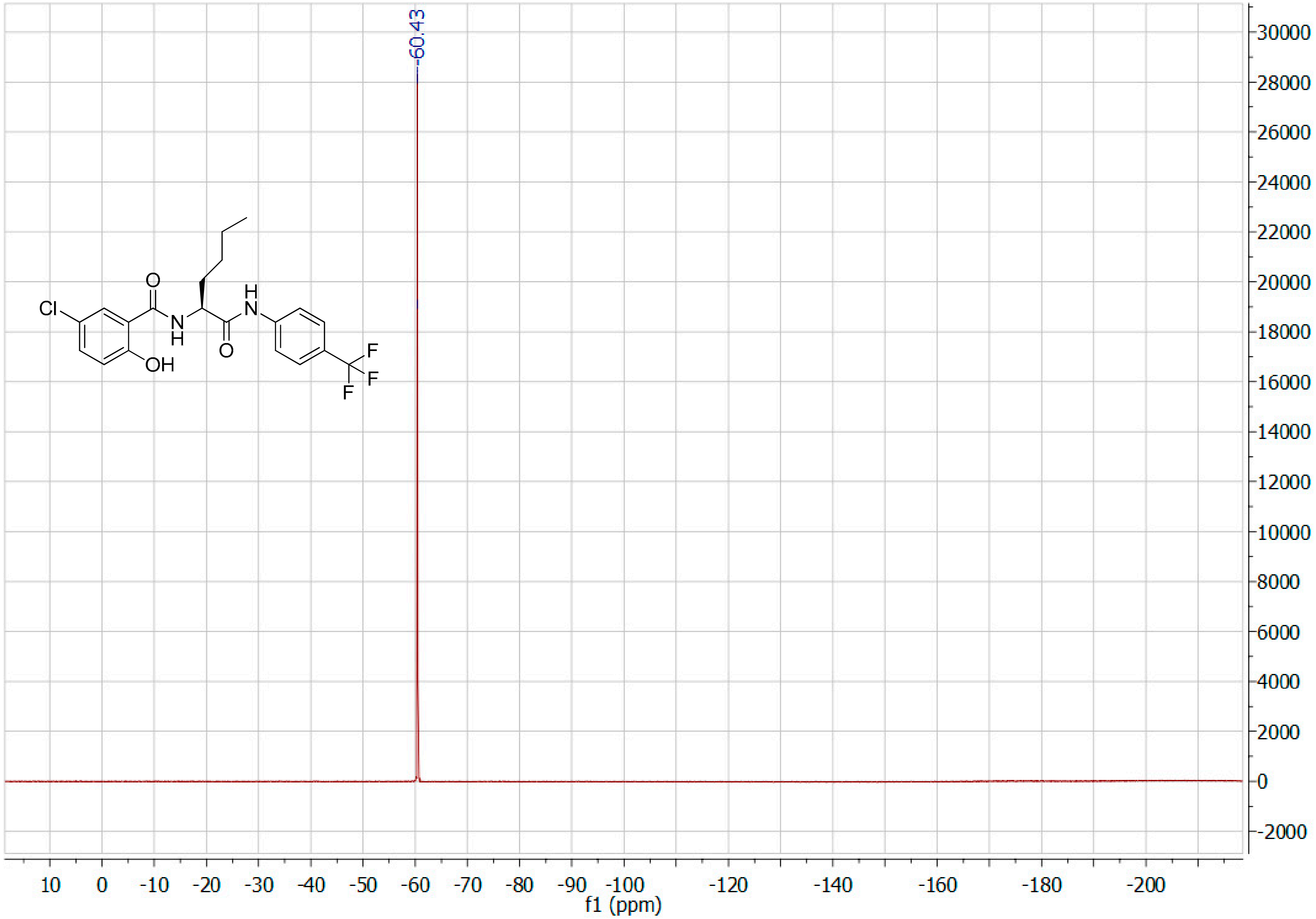

Figure S11. Copy of ^{1}H NMR 5-Chloro-2-hydroxy-*N*-[(2*S*)-4-(methylsulfanyl)-1-oxo-1-{[4-(trifluoromethyl)phenyl]amino}butan-2-yl]benzamide (**3e**)

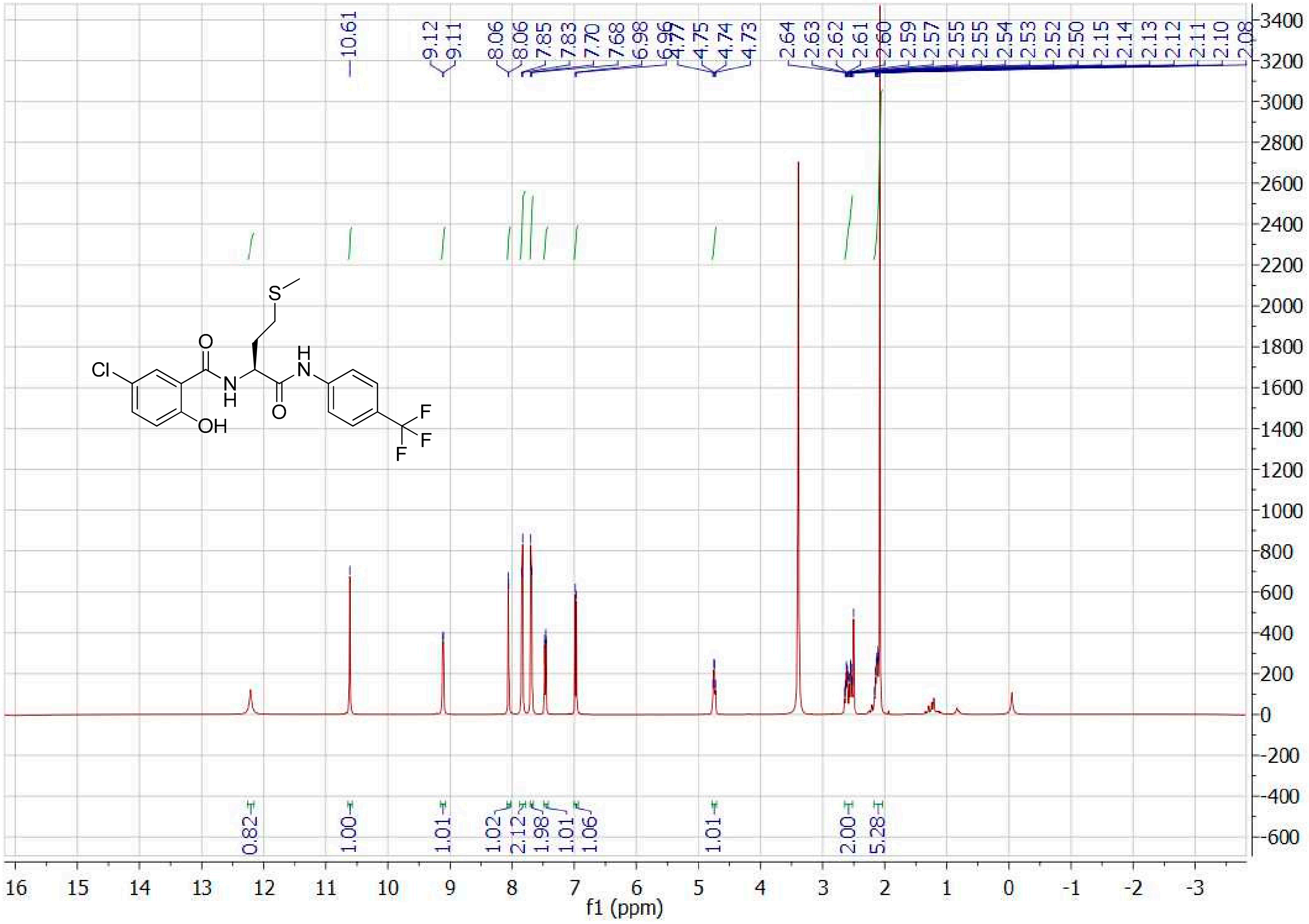

Figure S12. Copy of ^{13}C NMR 5-Chloro-2-hydroxy-*N*-[(2*S*)-4-(methylsulfanyl)-1-oxo-1-{[4-(trifluoromethyl)phenyl]amino}butan-2-yl]benzamide (**3e**)

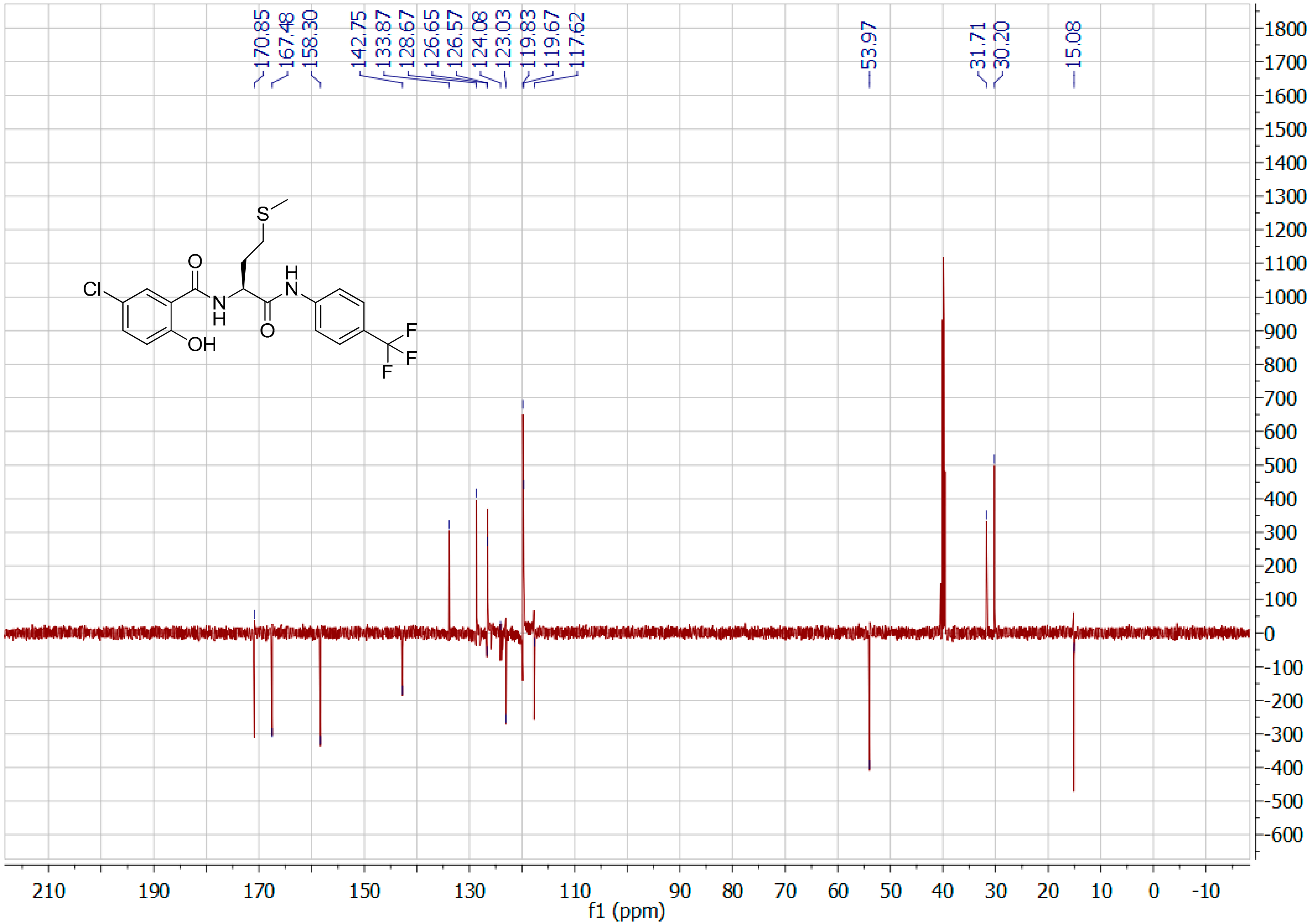

Figure S13. Copy of ^{19}F NMR 5-Chloro-2-hydroxy-*N*-[(2*S*)-4-(methylsulfanyl)-1-oxo-1-{[4-(trifluoromethyl)phenyl]amino}butan-2-yl]benzamide (**3e**)

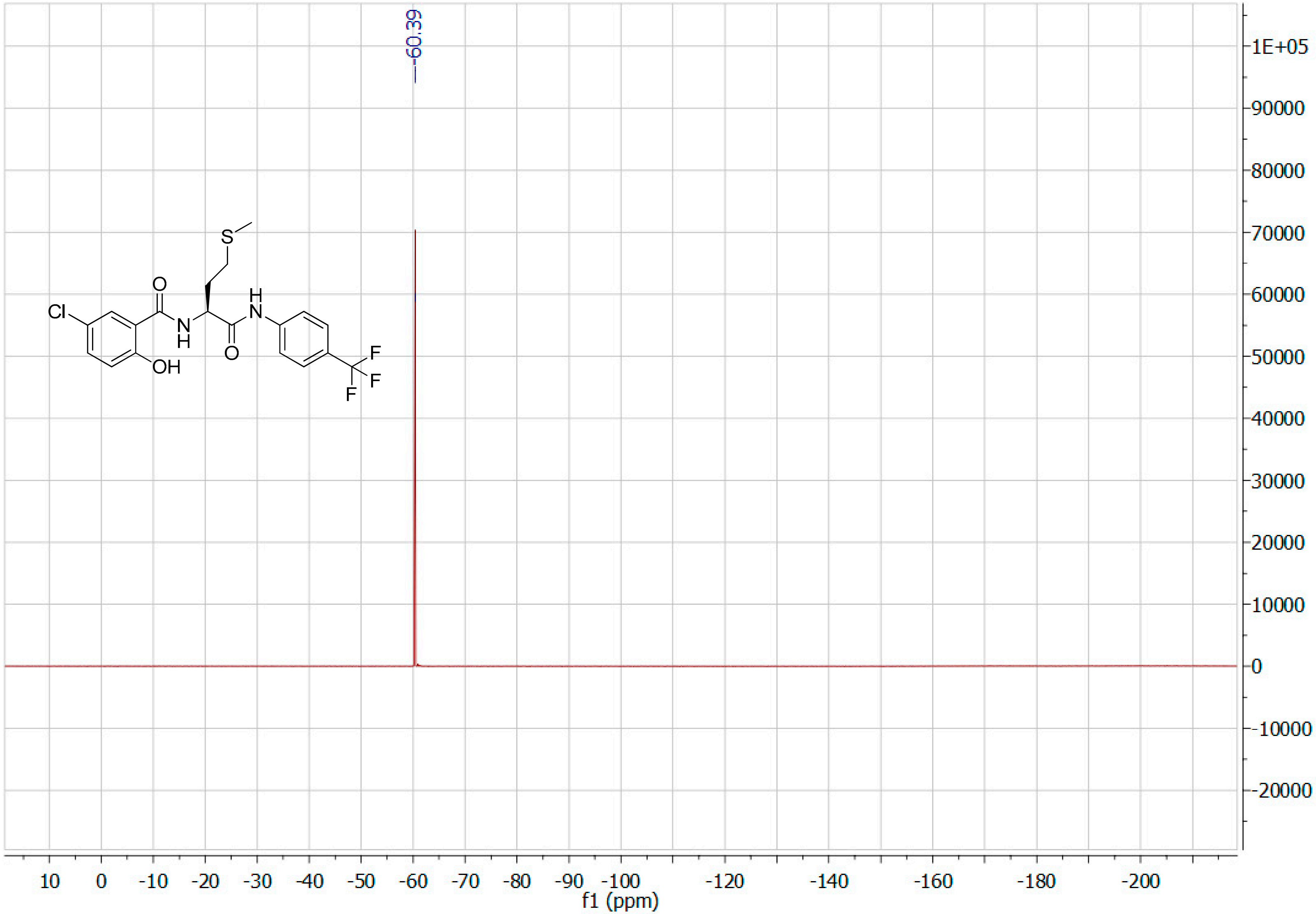

Figure S14. Copy of ^{1}H NMR 5-Chloro-*N*-[(2*S*)-3-cyclohexyl-1-oxo-1-{[4-(trifluoromethyl)phenyl]amino}propan-2-yl]-2-hydroxybenzamide (**3g**)]

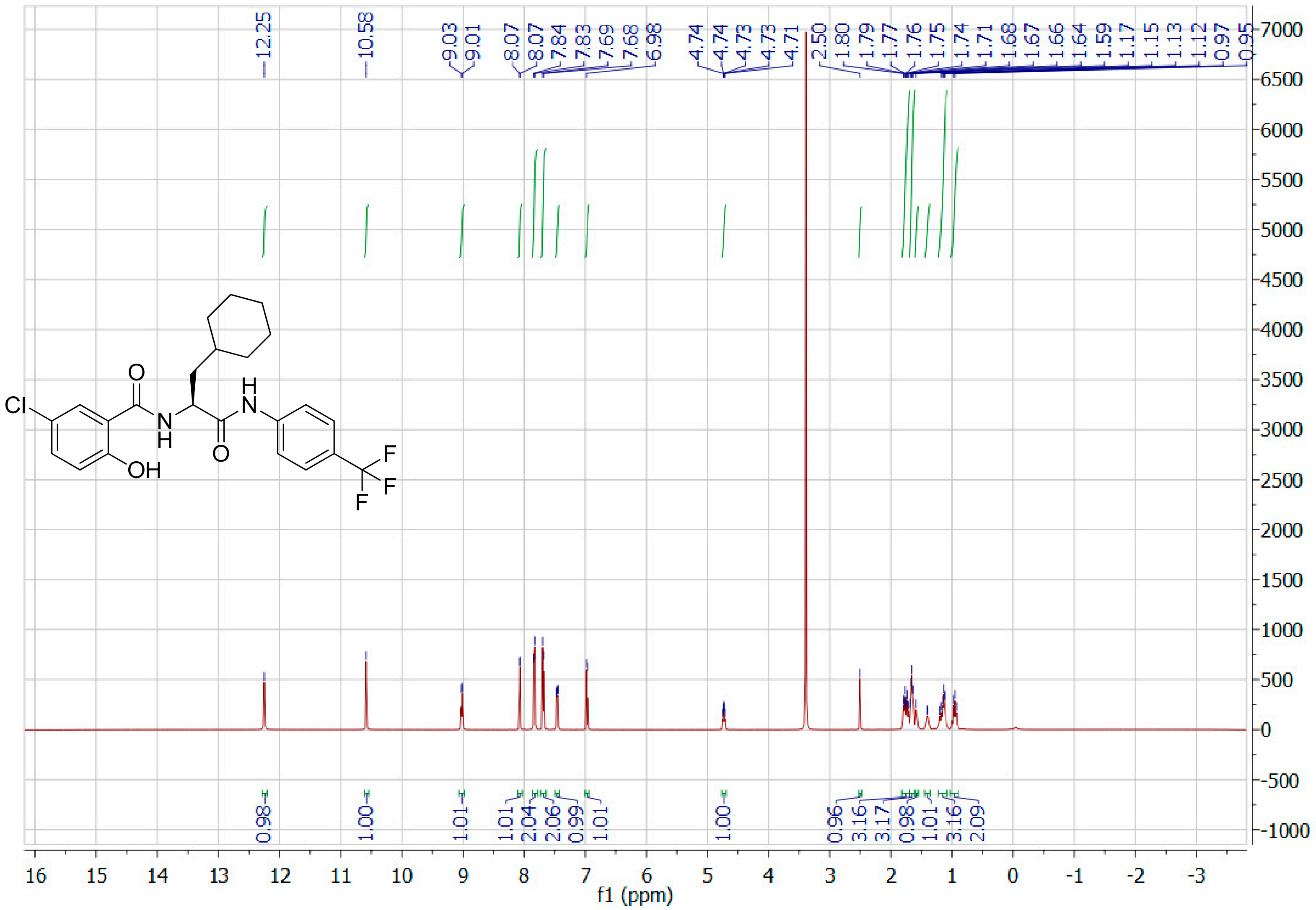

Figure S15. Copy of ^{13}C NMR 5-Chloro-*N*-[(2*S*)-3-cyclohexyl-1-oxo-1-{[4-(trifluoromethyl)phenyl]amino}propan-2-yl]-2-hydroxybenzamide (**3g**)

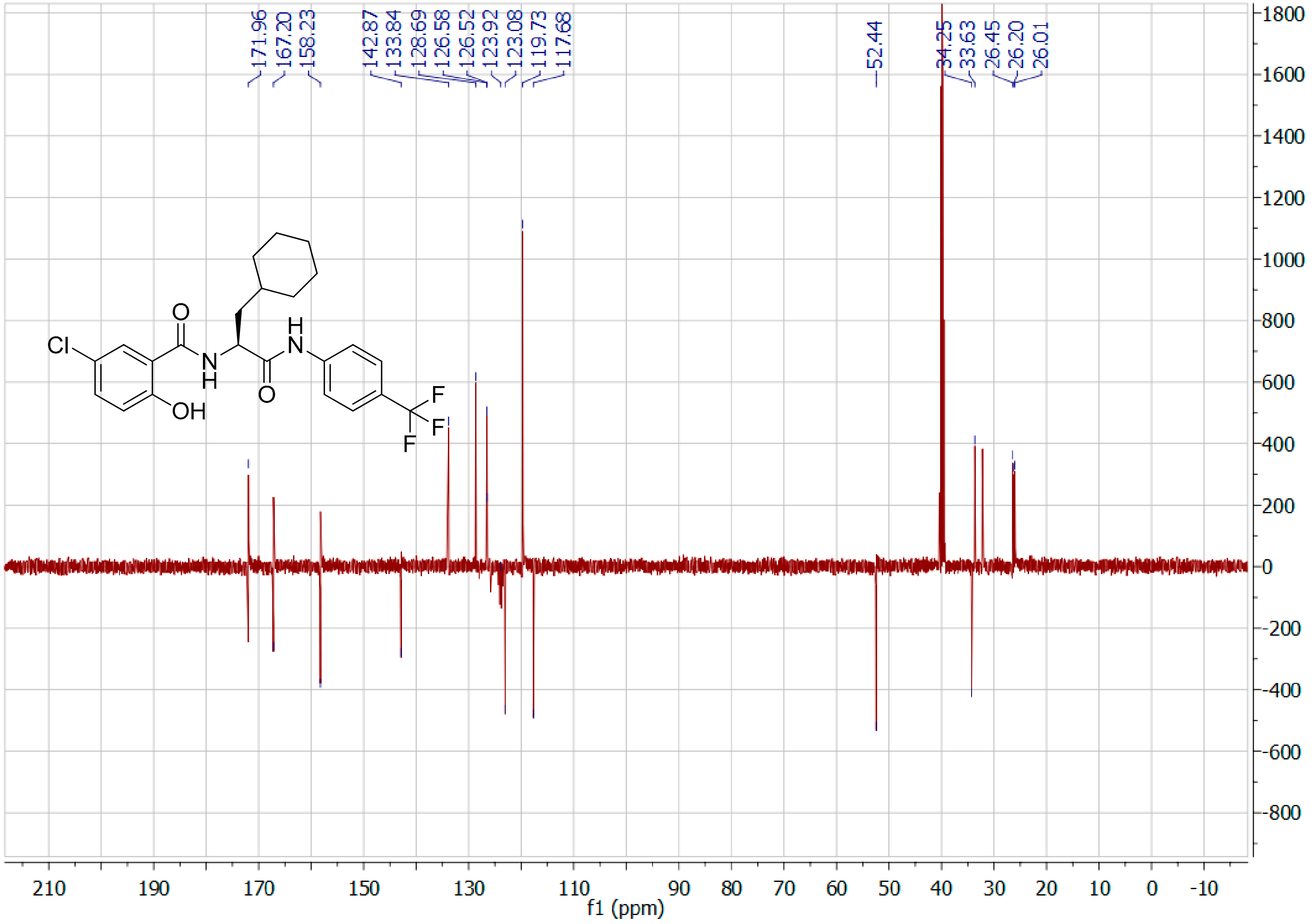

Figure S16. Copy of ^{19}F NMR 5-Chloro-*N*-[(2*S*)-3-cyclohexyl-1-oxo-1-{[4-(trifluoromethyl)phenyl]amino}propan-2-yl]-2-hydroxybenzamide (**3g**)

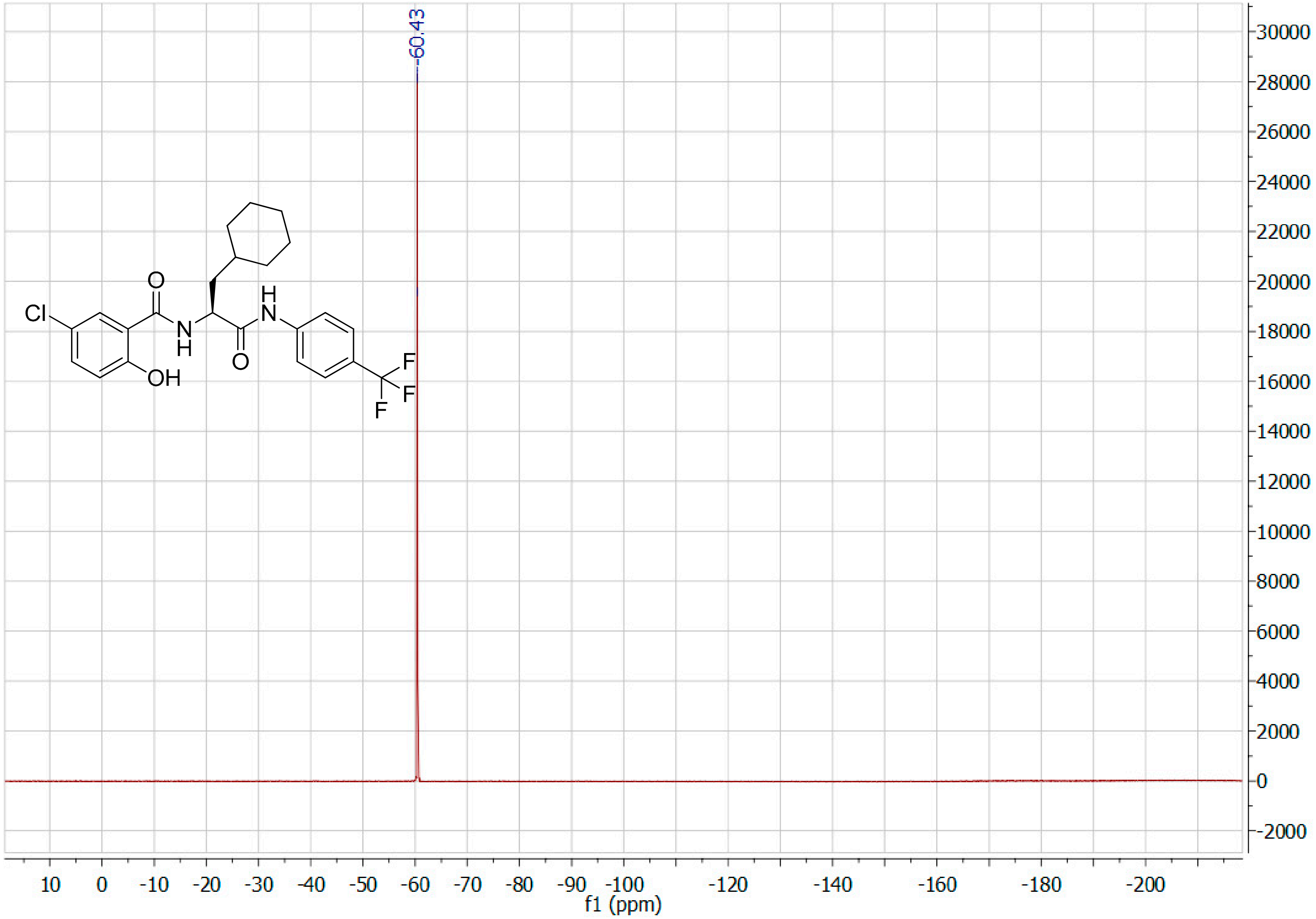

Supplement: Supplementary file 1 [file ijms-23-11648-s001.zip › ijms-1940637-supplementary.pdf]
